# The effectiveness of the McKenzie method in addition to first-line care for acute low back pain: a randomized controlled trial

**DOI:** 10.1186/1741-7015-8-10

**Published:** 2010-01-26

**Authors:** Luciana AC Machado, Chris G Maher, Rob D Herbert, Helen Clare, James H McAuley

**Affiliations:** 1 The George Institute for International Health, PO Box M201 Missenden Rd Sydney, NSW 2050, Australia; 2 Escola de Educação Física, Fisioterapia e Terapia Ocupacional, Universidade Federal de Minas Gerais, Av Antônio Carlos 6627, Pampulha, Belo Horizonte, MG 31270-901, Brazil; 3 Focus on Backs 1/124 Shirley Road, Crows Nest, NSW 2065, Sydney, Australia; 4 Faculty of Health Sciences, The University of Sydney, Sydney, Australia

## Abstract

**Background:**

Low back pain is a highly prevalent and disabling condition worldwide. Clinical guidelines for the management of patients with acute low back pain recommend first-line treatment consisting of advice, reassurance and simple analgesics. Exercise is also commonly prescribed to these patients. The primary aim of this study was to evaluate the short-term effect of adding the McKenzie method to the first-line care of patients with acute low back pain.

**Methods:**

A multi-centre randomized controlled trial with a 3-month follow-up was conducted between September 2005 and June 2008. Patients seeking care for acute non-specific low back pain from primary care medical practices were screened. Eligible participants were assigned to receive a treatment programme based on the McKenzie method and first-line care (advice, reassurance and time-contingent acetaminophen) or first-line care alone, for 3 weeks. Primary outcome measures included pain (0-10 Numeric Rating Scale) over the first seven days, pain at 1 week, pain at 3 weeks and global perceived effect (-5 to 5 scale) at 3 weeks. Treatment effects were estimated using linear mixed models.

**Results:**

One hundred and forty-eight participants were randomized into study groups, of whom 138 (93%) completed the last follow-up. The addition of the McKenzie method to first-line care produced statistically significant but small reductions in pain when compared to first-line care alone: mean of -0.4 points (95% confidence interval, -0.8 to -0.1) at 1 week, -0.7 points (95% confidence interval, -1.2 to -0.1) at 3 weeks, and -0.3 points (95% confidence interval, -0.5 to -0.0) over the first 7 days. Patients receiving the McKenzie method did not show additional effects on global perceived effect, disability, function or on the risk of persistent symptoms. These patients sought less additional health care than those receiving only first-line care (*P *= 0.002).

**Conclusions:**

When added to the currently recommended first-line care of acute low back pain, a treatment programme based on the McKenzie method does not produce appreciable additional short-term improvements in pain, disability, function or global perceived effect. However, the McKenzie method seems to reduce health utilization although it does not reduce patient's risk of developing persistent symptoms.

**Trial Registration:**

Australian New Zealand Clinical Trials Registry: ACTRN12605000032651

## Background

Current clinical guidelines [1-3] recommend the provision of advice, reassurance and simple analgesics as first-line treatment for patients with acute low back pain consulting a primary care physician. Although not recommended in most guidelines, exercises are also commonly prescribed for this population. In the USA over two-thirds of physicians include exercises in their initial care recommendations for patients with acute low back pain [4].

The effectiveness of exercises for the treatment of patients with acute low back pain is disputed. While many trials have concluded that exercises are ineffective for this population [5-7], these negative findings have been challenged on the grounds that the therapy was not appropriately administered [8]. A common criticism is that patients are asked to exercise in a way that does not take consideration of symptom response. Proponents of exercise therapy argue that better results would be obtained if exercises were customized to a patient's clinical presentation [8-11]. This treatment rationale forms the basis of the McKenzie method [12,13], which consists of a system of classification and classification-based treatment that is commonly used to treat low back pain in many countries and particularly in the USA [14] and Europe [15-17].

**Figure 1:**
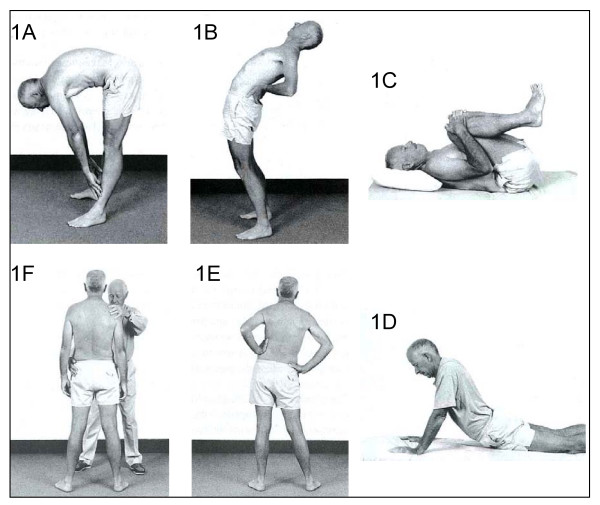
**Dynamic loading strategies applied to the spine in the McKenzie method: (A) flexion in standing; (B) extension in standing; (C) flexion in lying; (D) extension in lying; (E) side glide in standing; (F) therapist-assisted side glide in standing**. Reproduced with permission of Spinal Publications NZ Ltd.

Classification in the McKenzie method follows a comprehensive clinical examination including examination of posture and range of movement, together with the assessment of patient's symptomatic response to different loading strategies applied to the spine (Figure 1). Findings from this examination determine the classification of low back pain into one of three syndromes: derangement syndrome; dysfunction syndrome; or postural syndrome [12]. The core component of treatment in the McKenzie method is exercise, which consists of sustained postures or repeated movements similar to the loading strategies used for the assessment. This method also includes other components such as education and postural training.

With the McKenzie approach exercise is not used to strengthen the back muscles, but to promote rapid symptom relief. A key principle is to teach the patient simple strategies to self-manage their pain. For example, one pattern of symptomatic response observed during the execution of the different loading strategies is known as 'centralization' and describes the phenomenon by which pain referred from the spine is progressively abolished [12], and this can be observed as early as at the first visit to the therapist [18]. For a more detailed account of the full clinical picture of each of the syndromes and their respective treatment program, see McKenzie's textbooks [12,13].

The scientific evidence to support the use of the McKenzie method is still scarce, particularly in primary medical care. Only one high-quality randomized controlled trial, conducted by Cherkin *et al*. [19], has evaluated the effectiveness of the McKenzie method for patients with low back pain seeking care from primary care physicians. Cherkin's trial compared the McKenzie treatment to an educational booklet and found that the former was only marginally more effective for patients with predominantly acute low back pain who were refractory to the physician's care after 1 week [19]. No previous trial has evaluated the effectiveness of a treatment program based on the McKenzie method for patients with acute low back pain when they first presented to a primary care physician. We conducted a randomized controlled trial in which patients with acute low back pain first presenting to a primary care physician were randomized either to receive first-line care alone or first-line care and a treatment program based on the McKenzie method.

## Methods

This study was a multi-centre randomized controlled trial conducted between September 2005 and June 2008. The study was approved by the University of Sydney Human Research Ethics Committee and prospectively registered with the Australian and New Zealand Clinical Trials Registry (ACTRN12605000032651) [20].

### Patients

Thirty-one primary care physicians in 27 medical practices located in a socio-economically diverse region in Sydney, Australia, screened for eligibility consecutive patients seeking care for low back pain. The screening for eligible patients occurred between September 2005 and December 2007. To be eligible for inclusion, patients had to be 18 to 80 years old, present with a new episode of acute non-specific low back pain and be able and willing to visit one of the trial physical therapists for commencement of the McKenzie treatment program within 48 h of presentation to the physician. A new episode of acute non-specific low back pain was defined as pain in the area between the 12th rib and buttock crease (with or without leg pain) of less than 6 weeks duration, preceded by a period of at least 1 month without low back pain in which the patient did not consult a health care practitioner [21]. Patients were excluded if they had any of the following: nerve root compromise; 'red flags' for serious spinal pathology (for example, infection, fracture); spinal surgery in the past 6 months; pregnancy; severe cardiovascular or metabolic disease; or the inability to read and understand English. Written consent was obtained from all those who agreed to participate.

### Randomization

A statistician not involved in recruitment, data collection or treatment, developed a computer-generated randomization sequence (randomly permuted blocks of 4, 6 and 8) that was placed in sequentially numbered, sealed opaque envelopes [22]. All patients who satisfied the eligibility criteria and volunteered to participate were randomized to the First-line Care Group (recommended first-line care alone) or the McKenzie Group (McKenzie method in addition to the first-line care). It was not feasible to blind participants or therapists to the treatment allocation. Participants were informed that they would receive the best available care according to the current scientific knowledge and that they would have a 50% chance of receiving an additional exercise programme of unknown efficacy.

### Intervention

During the intervention period (3 weeks), participants were asked not to seek treatment for their back complaints other than that provided in the trial.

#### First-Line Care

Participants in the First Line Care Group received first-line care as per guideline recommendations [1-3]. As not all physicians were familiar with these recommendations, the physicians underwent individual training sessions with members of the research team (LM and JM) prior to the start of their participation in the study. The first-line care consisted of the provision of advice to remain active and to avoid bed rest, reassurance of the favourable prognosis of acute low back pain and instructions to take acetaminophen (paracetamol) on a time-contingent basis. Non-steroidal anti-inflammatory drugs (NSAIDs) were not prescribed during the ensuing 3 weeks. However, participants already on a course of NSAIDs when first visiting the primary care physician were allowed to continue use of this medication. Participants were instructed to follow the physician's advice for the next 3 weeks and, if necessary, to return for follow-up visits during this period. Although there was no limit to the number of follow-up visits, physicians were instructed to restrict treatment to advice and simple analgesics.

#### McKenzie method

In addition to the first-line care, participants in the McKenzie Group were immediately referred to a physical therapist and started a treatment programme based on the McKenzie method within 48 h of their consultation with the physician. Treatment was provided by 15 physical therapists who had completed at least 100 h of postgraduate training and had achieved the status of accredited (credentialed) McKenzie therapists. In order to ensure an optimal implementation of the treatment, physical therapists also attended a training session with a senior educator from the McKenzie Institute International (HC) prior to the commencement of the study.

Physical therapists were instructed to follow exclusively the treatment principles described in McKenzie's textbooks [12,13] and not to use other treatment modalities. After testing the participants' pain response to a comprehensive physical examination, therapists initially classified each patient into one of the three McKenzie syndromes (derangement, dysfunction, or postural) and an individualized treatment programme matching this classification was then provided. (For a brief description of syndromes and their matching treatment program see [20].) For most participants, the guiding treatment principle was to encourage directions of movement and postures that produced centralization of pain.

The number of treatment sessions was at the discretion of the physical therapist, with a maximum of six sessions over 3 weeks. In addition to the scheduled treatment sessions, participants were encouraged to perform the prescribed exercises at home and to follow the therapist's postural advice at all times. A copy of the *Treat Your Own Back *book [23] was provided to all participants, as usual in treatment with the McKenzie method. Some participants also received a lumbar support (original McKenzie lumbar roll) at the therapist's discretion. The McKenzie method was provided at no cost to participants.

### Outcomes

Our primary focus was on the short-term treatment effects because a treatment programme based on the McKenzie method is promoted as providing rapid symptom improvement in patients with low back pain [24-26]. Primary and secondary outcome measures were nominated *a priori*[20] and were self-assessed by participants.

#### Primary outcome measures

• Pain at 1 week, mean pain over the first 7 days and pain at 3 weeks: participants were asked to rate the average pain over the past 24 h on a 0-10 Numeric Rating Scale [27].

• Global perceived effect at 3 weeks: participants were asked to rate global perceived effect on a -5 to 5 scale, anchored at 'vastly worse' and 'completely recovered' [28].

#### Secondary outcome measures

• Disability at 1 and 3 weeks: participants were asked to measure any disability due to low back pain on the 0-24 Roland Morris Disability Questionnaire [29].

• Function at 1 and 3 weeks: participants were asked to measure function on a 0-10 Patient Specific Functional Scale [30].

• Global perceived effect at 1 week: same as global perceived effect at 3 weeks.

• Persistent low back pain at 3 months: participants were asked 'During the past 3 months have you ever been completely free of low back pain? By this I mean no low back pain at all, and would this pain-free period have lasted for a whole month'. Those answering 'No' were considered to have persistent low back pain.

### Adherence

Physical therapists assessed adherence with home exercises and postural correction for participants in the McKenzie Group at each visit. In order to be considered as being adherent, participants had to report they were maintaining the postural correction and performing the prescribed exercises at home on at least 50% of the consultations where adherence was assessed.

### Assessment procedure

Participants received an assessment booklet in which self-assessed outcomes were recorded. Within 24 h of the consultation with the primary care physician, and prior to randomization, a member of the research team contacted participants by telephone in order to provide instructions on how to complete the booklet and to collect demographic data. In order to reduce the potential for missing data due to participants misplacing outcome diaries, answers from the outcome diaries were transcribed by a researcher who was blinded to allocation during a telephone follow-up at 1 and 3 weeks (LM). At 3 months, the same blinded researcher contacted all participants to enquire about persistent low back pain and to collect information on additional health care services with respect to low back pain accessed after the 3-week treatment period.

### Statistical analysis

The sample size of 148 participants, determined *a priori*, provided better than an 80% power to detect a difference of 1 unit in pain scores [27] between treatment groups with an α level of 0.05, assuming a standard deviation [SD] of 2 units [31] and allowing for a loss to follow-up of up to 15%. This sample size also allowed for the detection of a difference of 1.2 units on the -5 to 5 global perceived effect scale (SD 2.4). Data were double-entered and analysed by intention-to-treat.

Treatment effects were estimated using linear mixed models (random intercept and fixed coefficients) which incorporated treatment, time and the interaction between treatment and time. Outcomes were linearly related to the log of time (*r*^2 ^of mean outcomes versus log time ≥ 0.97) so time was entered into the models as the log of time. Analyses were conducted with the 'xtmixed' procedure in Stata v9. Estimates of effects at specific time points (1 and 3 weeks) and the mean effect on pain over the first 7 days were obtained from the regression model using the 'lincom' routine. Relative risks were calculated from the ratio of the proportions of participants in the two groups with persistent symptoms and the use of other health care treatments.

## Results

**Figure 2:**
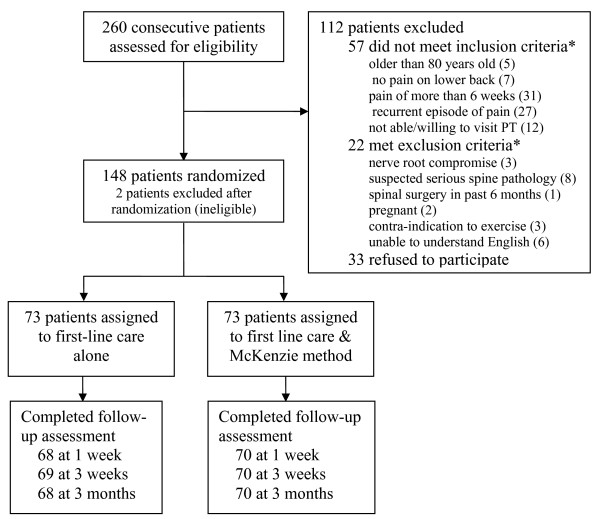
**Flow of participants through the trial**. *Some patients presented more than one exclusion criteria.

Between September 2005 and December 2007, primary care physicians screened 260 consecutive patients for eligibility. A total of 148 patients were randomized (Figure 2). One patient randomized to each group was misdiagnosed. Both were subsequently ruled ineligible to participate in the trial and, thus, were considered legitimate post-randomization exclusions [32] (one had pain from kidney stones and the other had pain in the thoracic spine only). The two groups' demographic and clinical characteristics were similar at baseline (Table 1).

**Table 1:** Characteristics of participants at baseline.

	McKenzie (*N *= 73)	First-line Care Group (*N *= 73)
Age	47.5 ± 14.4	45.9 ± 14.9
Sex (female)	38 (52%)	35 (48%)
Duration of current low back pain episode		
Less than 2 weeks	48 (66%)	49 (67%)
From 2 to 6 weeks	25 (34%)	24 (33%)
Pain radiating to the leg	33 (45%)	36 (50%)
Pain is movement-dependant*	61 (84%)	58 (80%)
Previous episode of low back pain	54 (74%)	49 (67%)
Participating in moderate exercise^†^	44 (60%)	46 (63%)
Taking medication (any types)	54 (74%)	52 (71%)
Using non-steroidal anti-inflammatory drugs	28 (38%)	22 (30%)
Days off work or school due to low back pain	0.7 ± 1.4	0.8 ± 1.2
Compensation case (worker's compensation)	3 (4%)	1 (1%)
General health status		
Excellent	11 (15%)	16 (22%)
Very good	35 (48%)	37 (51%)
Good	18 (25%)	17 (23%)
Fair	6 (8%)	2 (3%)
Poor	3 (4%)	1 (1%)
Pain^‡^	6.6 ± 1.8	6.3 ± 1.9
Disability^§^	13.7 ± 5.5	13.5 ± 5.3
Function^¶^	3.7 ± 1.6	3.4 ± 1.8

Data are means ± standard deviations or frequencies (%).

*Positive answer to question 'Does your pain change in intensity or location depending on any position or movement?'.

^†^Moderate exercise was any type of exercise performed for at least 30 min, three times per week or more.

^‡^Numerical rating scale, scored from 0 (no pain) to 10 (worst pain possible).

^§^Roland-Morris Disability Questionnaire, scored from 0 (no disability) to 24 (high disability).

^¶^Patient Specific Functional Scale, scored from 0 (unable to perform activity) to 10 (able to perform activity at pre-injury level).

NSAIDs, non-steroidal anti-inflammatory drugs.

Of the participants allocated to the McKenzie Group, 94% were initially classified into the derangement syndrome and 6% were classified into the dysfunction syndrome. Lumbar rolls were prescribed to 93% of participants. Participants received a median of four (range 1-6) sessions with the physical therapist over a 3-week period, with a median of two (range 1-3) sessions over the first week. Data on exercise adherence over the first week were available for 56 participants in the McKenzie Group and data on exercise adherence over the 3-week treatment period were available for 50 participants in the McKenzie Group. Adherence rates (proportions) were 66% over the first week and 74% over the treatment period. The group for which adherence data was available had similar baseline scores for pain, disability and function to the group for whom data was not available (data not shown). The maximum number of participants lost to follow-up at any time-point was eight (5%).

**Figure 3:**
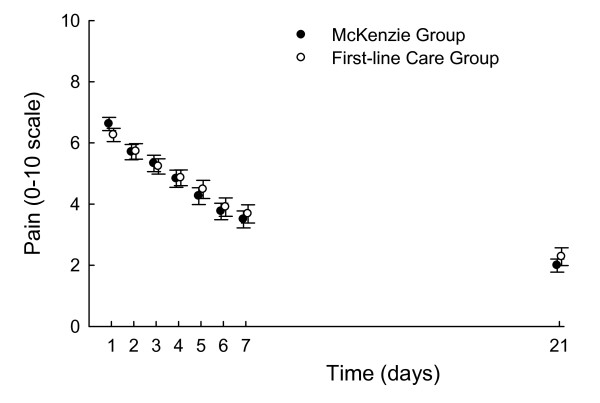
**Pain scores in the McKenzie and First-line Care groups**. Values are unadjusted means and standard errors. For clarity, data for the two groups have been slightly offset on the time axis.

Table 2 shows the mean outcomes by group and Figure 3 shows reductions in pain over time in the groups. The additional effects of the McKenzie method on pain (that is, the adjusted difference in outcomes between the McKenzie Group and the First-line Care Group) were statistically significant but smaller than our pre-specified threshold for between-group clinical importance of 1 unit (*P *= 0.02; Table 2). The addition of the McKenzie method reduced pain by a mean of 0.4 points on a 0-10 pain scale at 1 week (95% confidence interval [CI], -0.8 to -0.1) and by a mean of 0.7 points at 3 weeks (95% CI, -1.2 to -0.1). The average pain experienced over the first 7 days was also slightly lower in the McKenzie Group (mean effect, -0.3; 95% CI, -0.5 to -0.0). For all other outcomes, the additional effects of the McKenzie method were near zero at all time points and not statistically significant (Table 2).

**Table 2:** Mean outcomes in treatment groups and effects of the addition of the McKenzie method to the recommended first-line care.

Outcome	N**	Unadjusted mean outcome (SE)		Adjusted mean outcome (SE)		Treatment effect* (95% CI)	*P *value
		**McKenzie**	**First-line Care**	**McKenzie**	**First-line Care**	**McKenzie - First-line Care**	
			
**Pain**^**†**^							*0.02*
*1 week*	*70/69*	*3.5 (0.3)*	*3.7 (0.3)*	*3.6 (0.2)*	*4.0 (0.2)*	*-0.4 (-0.8 to -0.1)*	
*3 weeks*	*70/68*	*2.0 (0.2)*	*2.3 (0.3)*	*1.8 (0.2)*	*2.5 (0.2)*	*-0.7 (-1.2 to -0.1)*	
*Mean pain over first 7 days*	*70/69*	*4.9 (0.2)*	*4.9 (0.2)*	*4.7 (0.2)*	*5.0 (0.2)*	*-0.3 (-0.5 to -0.0)*	
**Global perceived effect**^**‡**^							
1 week	70/68	2.6 (0.2)	2.1 (0.2)	2.6 (0.2)	2.1 (0.2)	0.5 (-0.0 to 1.1)	0.07
*3 weeks*	*70/69*	*3.6 (0.1)*	*3.3 (0.2)*	*3.6 (0.1)*	*3.3 (0.2)*	*0.3 (-0.3 to 0.8)*	*0.33*
**Disability**^**§**^							0.74
1 week	70/68	8.4 (0.7)	9.0 (0.8)	8.0 (0.5)	8.2 (0.5)	-0.2 (-1.5 to 1.0)	
3 weeks	70/69	4.6 (0.7)	4.5 (0.7)	4.8 (0.7)	5.1 (0.7)	-0.3 (-2.3 to 1.6)	
**Function**^**¶**^							0.90
1 week	70/68	6.2 (0.3)	5.8 (0.3)	6.2 (0.2)	6.2 (0.2)	0.0 (-0.4 to 0.5)	
3 weeks	70/69	7.9 (0.2)	7.7 (0.3)	7.8 (0.2)	7.7 (0.3)	0.0 (-0.7 to 0.8)	

*Treatment effects are model-based adjusted differences in outcomes between groups. For a global perceived effect, which was measured only at two time points after randomization, adjusted means and treatment effects are the same as unadjusted means and treatment effects. Primary outcomes are highlighted in italics. Effectiveness of the addition of the McKenzie method is indicated by negative effects for pain and disability and by positive effects of global perceived effect and function. **Number of participants in McKenzie/First-line Care groups for whom data were available.

^†^Numerical rating scale, scored from 0 (no pain) to 10 (worst pain possible).

^‡^Global perceived effect scale scored from -5 (much worse) to 5 (completely recovered). ^§^Roland-Morris Disability Questionnaire, scored from 0 (no disability) to 24 (high disability).

^¶^Patient Specific Functional Scale, scored from 0 (unable to perform activity) to 10 (able to perform activity at pre-injury level).

SE, standard error.

CI, confidence interval.

Thirty-seven (53%) subjects in the McKenzie Group and 32 (47%) in the First-line Care Group developed persistent low back pain; this difference was not statistically significant (relative risk, 1.1; 95% CI, 0.8 to 1.6; *P *= 0.49). Subjects under treatment with the McKenzie method were less likely to seek additional health care for their back complaints after the 3-week treatment period; five participants (7%) in the McKenzie Group and 18 participants (26%) in the First-line Care Group sought additional health care (relative risk, 0.27; 95% CI, 0.1 to 0.7; *P *= 0.002). According to the participants' reports, the most commonly sought additional treatments were other forms of physical therapy (32%), NSAIDs (18%) and acupuncture (14%).

## Discussion

Findings of our study are in line with current clinical practice guidelines, which support the view that most patients with acute non-specific low back pain will exhibit a speedy recovery. For instance, participants from both the McKenzie Group and the First-line Care Group showed large short-term improvements in outcomes from baseline (for example, there were 53% and 59% reductions in pain at 1 week follow-up, respectively). However, our trial showed that, in patients with acute low back pain receiving recommended first-line care, the addition of a treatment programme based on the McKenzie method did not have clinically meaningful effects on pain, disability, function, global perceived effect or risk of developing persistent symptoms.

We considered the additional effect of the McKenzie method on pain (that is, adjusted between-group differences in pain of 0.7 points or less) to be small. For instance, this effect was less than that considered by a panel of experts to be a minimal important change [33]. We acknowledge that cut-offs used to detect minimal important changes are generally developed to assist interpretation of within-group differences. Nevertheless, we still believe the magnitude of the additional effect of the McKenzie method on pain was trivial in our trial and would not be considered worthwhile by most health practitioners responsible for managing patients with acute low back pain in primary care and, possibly, also by the patients. In making this judgement we also took into account the fact that participants and therapists were not (and could not be) blinded to treatment allocation. Lack of blinding is likely to exaggerate treatment effects on subjective outcomes such as pain [34]. Finally, estimates of the effects of treatments in the present trial were very precise, as indicated by the narrow confidence intervals (it is unlikely that improvements seen after the addition of the McKenzie method to first-line care would be larger than 12% even in the best-case scenario).

Some trials provide a description of the results by computing the proportion of subjects who improve with each treatment. We chose not to follow this approach in the current trial because statistical power is reduced when continuous outcomes are dichotomized and because the choice of cut-offs for improvement can influence the results. As an illustration for pain improvement it was seen that at 1 week, with a cut-off of two points or less for improvement, the McKenzie Group had more participants who improved (54.4% versus 45.6%), when the cut-off for improvement was raised to three or four points the First-line Care Group had more participants who improved (53.7% versus 46.3%); and for a cut-off of five or six points there was a similar number of participants improved in both groups (50%). This pattern demonstrates the limitations of this approach.

The addition of the McKenzie method to first-line care may be worthwhile under the health sector perspective. This is because we found that participants in the First-line Care Group sought more additional care than participants in the McKenzie Group. Unfortunately, this assumption cannot be confirmed from the results of the present trial since a proper cost-effectiveness analysis would need to be conducted and our trial was not designed to include such analysis (our main focus was on treatment effectiveness). It is possible that the imbalance found in the usage of additional care could have diluted the estimates of the effect of the McKenzie method at 3 months. However, in our view, the greater use of additional care by those in the First-line Care Group does not significantly threaten the internal validity of our study because our primary interest was in the effects of the McKenzie method in the first 3 weeks. Moreover, the available evidence from randomized trials suggests that the effects of other treatments for acute low back pain are modest at best [1,7] and so any additional care is unlikely to have substantially distorted estimates of effects of the McKenzie method at 3 months.

The quality of complex interventions, such as exercise, probably influences the size of the effect observed in randomized controlled trials [35]. In our trial the McKenzie method was applied by therapists who had undergone many hours of postgraduate training provided by the McKenzie Institute International and were considered to have expertise in the provision of this therapy [36]. Thus, it is doubtful that other therapists would have been likely to deliver the treatment with greater fidelity to McKenzie's principles. Likewise, because physicians in this trial were educated about current guideline-based recommendations, it is possible that the first-line care they provided differs from that typically provided by primary care physicians. Data from recent surveys show that many physicians are not familiar with the recommendations of clinical guidelines or do not usually follow guideline recommendations [37,38].

Although previous randomized controlled trials [19,39,40] have attempted to investigate the effects of a treatment programme based on the McKenzie method for low back pain in primary medical care, only the trial conducted by Cherkin *et al*. [19] has implemented this therapy according to its original principles (that is, the programme was individualized and properly customized to the patient's clinical presentation) [12,41]. Cherkin *et al*. [19] found that an individualized and customized treatment programme based on the McKenzie method reduced patient's 'bothersomeness of symptoms' by 0.8 points on a 0-10 bothersomeness scale at 4 weeks, compared to an educational booklet. This effect was not statistically significant at the conventional 0.05 level (*P *= 0.06). Apart from a greater satisfaction with treatment among participants receiving the McKenzie method, the authors did not find any greater effect of this therapy on any other outcome at any time-point when compared to an educational booklet [19]. Our trial and the trial of Cherkin *et al*. [19] found remarkably similar estimates of the effects of the McKenzie method. Both trials support the conclusion that a treatment programme based on the McKenzie method does not provide an appreciable benefit for patients already receiving relatively simple, low-risk and inexpensive treatments, such as advice, reassurance and simple analgesics or an educational booklet.

Our trial has a number of strengths. The investigation of the additional effects of the McKenzie method in patients already receiving first-line care from the primary care physician is not common in the low back pain literature. However, such a study design provides information that is extremely relevant and helpful to physicians in their clinical decision making as current practice guidelines recommend that patients are only referred for additional treatment if they do not succeed with previous first-line care. In addition, the treatment in our trial was delivered by highly-trained therapists, patient's adherence to the treatment programme was satisfactory and we had a very low rate of loss to follow-up. We acknowledge that it would have been preferable to select only one primary outcome measure in order to minimize the potential for a type I error (when we registered the trial we specified four primary outcome measures). However, this would be a concern if we were claiming that the statistically significant pain outcomes were clinically important, whereas our view is that these effects are trivially small. One limitation was the lack of therapist and patient blinding that could have been possible with the use of a placebo. However, given the nature of the programme of the McKenzie therapy, therapist blinding was not feasible and, in the design stage, there was no consensus among the research team on a placebo that was both clearly inert and credible to the patient. Another limitation was that adherence to protocol by either the physical therapists or primary care physicians were not evaluated. Finally, the lack of a proper cost-effectiveness analysis and the short-term follow-up limited the scope of our conclusions.

## Conclusions

A treatment programme based on the McKenzie method does not produce appreciable improvements in pain, disability, function, global perceived effect or risk of developing persistent symptoms in patients with acute low back pain receiving recommended first-line care. Patients with acute low back pain receiving only the recommended first-line care seek more additional health care than patients receiving the McKenzie method.

## Abbreviations

CI: confidence interval; NSAIDs: non-steroidal anti-inflammatory drugs; SD: standard deviation.

## Competing interests

HC is the Director of Education for the McKenzie Institute International. There are no other potential competing interests to report.

## Authors' contributions

LACM, CGM, RDH, JHM and HC contributed to the study conception and design. LACM and JHM were responsible for the data acquisition. LACM, CGM and RDH performed the analysis and interpretation of data. LACM and CGM were responsible for drafting the manuscript. All authors performed a critical revision of the manuscript for important intellectual content. LACM, JHM and HC provided administrative and technical support. LACM was the study coordinator. All authors read and approved the final manuscript.

## Pre-publication history

The pre-publication history for this paper can be accessed here:

http://www.biomedcentral.com/1741-7015/8/10/prepub
